# Circumvention of Gefitinib Resistance by Repurposing Flunarizine via Histone Deacetylase Inhibition

**DOI:** 10.1021/acsptsci.3c00202

**Published:** 2023-09-28

**Authors:** Kenneth K. W. To, James C. H. Chow, Ka-Man Cheung, William C. S. Cho

**Affiliations:** † School of Pharmacy, Faculty of Medicine, The Chinese University of Hong Kong, Hong Kong, SAR, China; ‡ Department of Clinical Oncology, Queen Elizabeth Hospital, Hong Kong, SAR, China

**Keywords:** flunarizine, gefitinib, histone deacetylase inhibitor, non-small cell lung cancer, repurposing

## Abstract

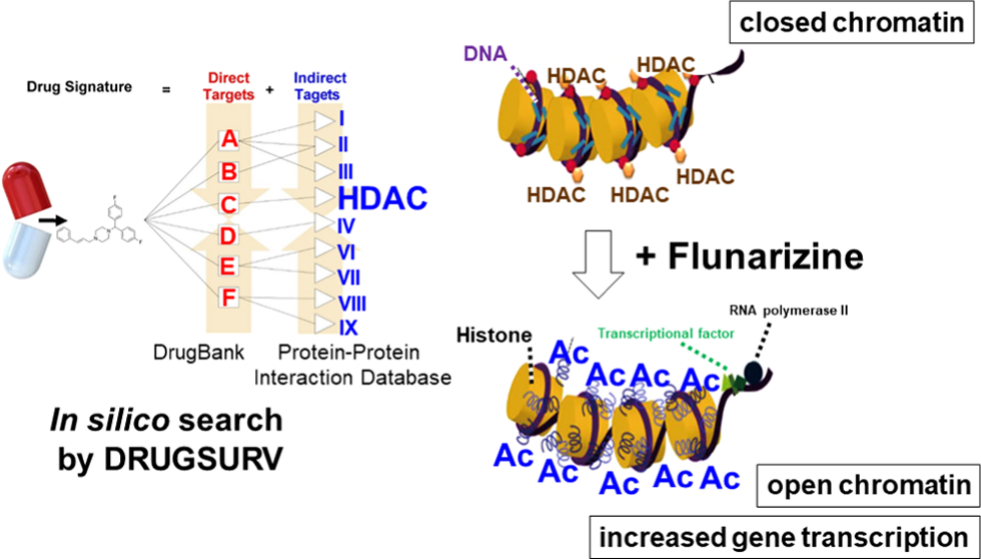

Gefitinib is an epidermal growth factor receptor tyrosine kinase inhibitor (EGFR TKI) for treating advanced non-small cell lung cancer (NSCLC). However, drug resistance seriously impedes the clinical efficacy of gefitinib. This study investigated the repositioning of the non-oncology drug capable of inhibiting histone deacetylases (HDACs) to overcome gefitinib resistance. A few drug candidates were identified using the in silico repurposing tool “DRUGSURV” and tested for HDAC inhibition. Flunarizine, originally indicated for migraine prophylaxis and vertigo treatment, was selected for detailed investigation in NSCLC cell lines harboring a range of different gefitinib resistance mechanisms (EGFR T790M, KRAS G12S, MET amplification, or PTEN loss). The circumvention of gefitinib resistance by flunarizine was further demonstrated in an EGFR TKI (erlotinib)-refractory patient-derived tumor xenograft (PDX) model in vivo. The acetylation level of cellular histone protein was increased by flunarizine in a concentration- and time-dependent manner. Among the NSCLC cell lines evaluated, the extent of gefitinib resistance circumvention by flunarizine was found to be the most pronounced in EGFR T790M-bearing H1975 cells. The gefitinib–flunarizine combination was shown to induce the apoptotic protein Bim but reduce the antiapoptotic protein Bcl-2, which apparently circumvented gefitinib resistance. The induction of Bim by flunarizine was accompanied by an increase in the histone acetylation and E2F1 interaction with the *BIM* gene promoter. Flunarizine was also found to upregulate E-cadherin but downregulate the vimentin expression, which subsequently inhibited cancer cell migration and invasion. Importantly, flunarizine was also shown to significantly potentiate the tumor growth suppressive effect of gefitinib in EGFR TKI-refractory PDX in vivo. The findings advocate for the translational application of flunarizine to circumvent gefitinib resistance in the clinic.

Lung cancer is the most common cause of cancer death worldwide.^1^ The prognosis of advanced non-small cell lung cancer (NSCLC), a histological subtype accounting for approximately 85% of all lung cancers, is very poor before the advent of immunotherapy and molecularly targeted therapies.^2^ Traditional cisplatin-based chemotherapy is the mainstay of treatment for advanced NSCLC, but it is notorious for severe toxicities.^3^ Recent advances in precision oncology research allowed the efficacious clinical management of a subset of NSCLC patients bearing specific epidermal growth factor receptor (EGFR) mutations by EGFR tyrosine kinase inhibitors (EGFR TKIs) with a favorable side effect profile. However, both innate and acquired resistances to EGFR TKIs significantly hinder their clinical outcome.^4^ The development of effective and tolerable regimens for circumventing EGFR TKI resistance is desperately needed.

Drug resistance to EGFR TKIs is multifactorial.^4^ Innate resistance to EGFR TKIs is mainly mediated by the KRAS mutation or PTEN loss, thereby bypassing EGFR inhibition by the TKIs. On the other hand, acquired resistance to EGFR TKIs is usually caused by the emergence of a secondary EGFR T790 M mutation. Dysregulation of other parallel oncogenic pathways, including *MET* amplification and *HER2* mutation, was also reported to cause EGFR TKI resistance. Novel treatment strategies are needed to address the unmet medical needs of NSCLC patients who have resistance to EGFR TKIs.

Histone deacetylases (HDACs) regulate the expression of oncogenes and tumor suppressor genes by dynamically modulating the level of histone acetylation in chromatin within the nucleus.^5^ Moreover, HDACs also regulate various nonhistone proteins important for cancer development.^6^ As HDACs are usually aberrantly regulated in drug refractory cancer, they represent novel therapeutic targets to overcome chemoresistance.^7^ To date, four HDAC inhibitors (HDACIs) (vorinostat (SAHA), romidepsin, belinostat, and panobinostat) are clinically approved for cancer treatment by major drug regulatory authorities.^7^ Drug combinations with HDACIs are considered a promising strategy to overcome chemoresistance.^8,9^ Extensive preclinical and clinical research about these novel drug combinations has been conducted with promising findings.^10,11^ HDACIs were reported to modulate pro-apoptotic and cell cycle regulatory genes to elicit their anticancer activities.^12^

Drug repurposing refers to the investigation of clinically approved drugs for new therapeutic indications. In recent years, this approach has attracted increasing interest for use in cancer therapy. The aim of this study was to identify new HDACIs that could overcome gefitinib resistance in NSCLC treatment both in vitro and in vivo. Flunarizine was identified as a novel HDACI by using the publicly accessible computational tool “DRUGSURV” from clinically approved non-oncology drugs. It was investigated in terms of its HDAC inhibitory effect to enhance the anticancer effect of gefitinib. The circumvention of gefitinib resistance by flunarizine was further corroborated in an EGFR TKI-refractory NSCLC patient-derived tumor xenograft (PDX) model.

## Results

### Clinically Approved Non-oncology Drugs Were Identified As New HDACIs

According to our in silico search using DRUGSURV, high levels of HDAC1, 2, or 6 were remarkably correlated with poor survival (*p* < 0.05) in GEO datasets (GSE31210, GSE11969, and GSE36471, respectively) of lung cancer patients. HDAC1, 2, and 6 were therefore queried using DRUGSURV in order to single out clinically approved drugs that may target these HDACs, thereby extending patient survival. A list of putative HDACIs was identified from clinically approved drugs, and 12 candidates with relatively higher bioavailability and lower tendency for drug–drug interactions were shortlisted for investigation (Table 1).

**Table 1:** HDACI Drug Candidates Identified by Using the Free-Source DRUGSURV Program^a^^,^^b^^,^^c^

drug candidate	original therapeutic indication(s)	indirect drug target(s) to mediate HDAC inhibition	putative HDAC isoform target(s)^c^
(1) alfuzosin	benign prostatic hyperplasia	EHMT2	HDAC2
(2) baclofen	muscle spasm, multiple sclerosis	NFKB1	HDAC1
(3) candesartan	hypertension	ERBB2	HDAC6
(4) cinnarizine	motion sickness	ESR1	HDAC1 and 2
(5) clemastine	allergy	TP53	HDAC1, 2, and 3
(6) danazol	endometrosis	ESR1; MYC	HDAC1 and 2
(7) disulfiram	alcohol deterrent	EHMT2	HDAC1
(8) flunarizine	prophylaxis of migraine	EHMT2	HDAC1 and 2
(9) guanfacine	attention deficit hyperactivity disorder	EHMT2	HDAC2
(10) raloxifene	osteoporosis	ESR1; FYN; TP53	HDAC3 and 6
(11) telmisartan	hypertension	EHMT2	HDAC1, 2, and 3
(12) triamterene	diuretic	ESR1; TP53	HDAC1, 2, and 3

a Twelve drug candidates are tabulated in alphabetical order. Original therapeutic indication(s); possible indirect mediator(s) to give rise to HDAC inhibition; and putative targeted HDAC isoform(s) are summarized.

b Abbreviations: EHMT2 = euchromatic histone-lysine *N*-methyltransferase 2; ESR1 = estrogen receptor 1; FYN = a Src family tyrosine kinase; MYC = c-myc oncoprotein; TP53 = tumor protein P53.

c Obtained from the “Gene Query” function of DRUGSURV.

**Figure 1:**
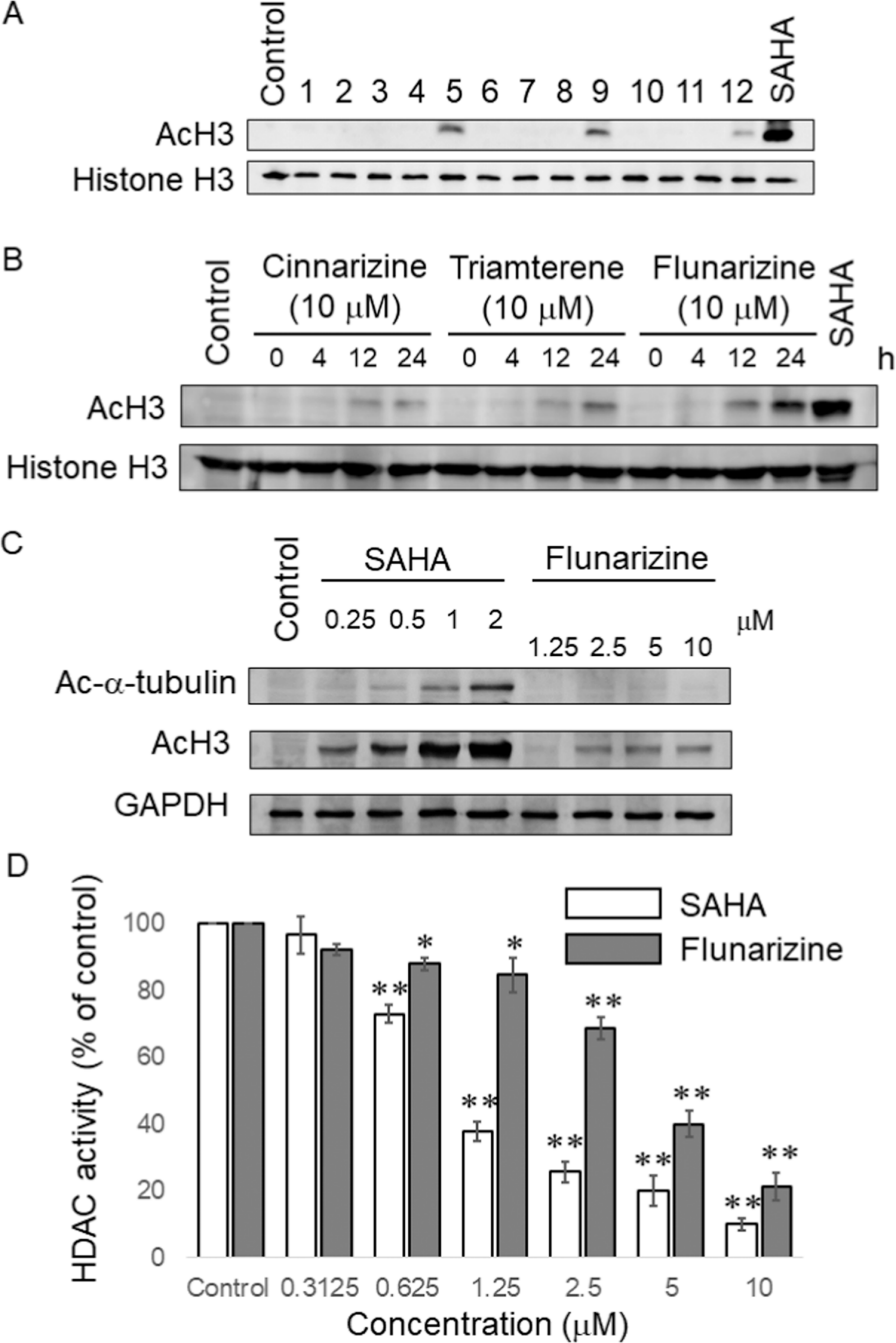
Flunarizine increased the acetylation level of histone H3 in a concentration- and time-dependent manner in H1975 cells harboring the EGFR T790 M mutation. (A) Western blot data demonstrating the increase in histone H3 acetylation (Lys 9,14) (AcH3) in H1975 cells after incubation with different drug candidates at 10 μM for 24 h. SAHA (2 μM) was used as a control for comparison. Total histone H3 protein expression was used as the loading control. Drug candidates 1–12 are alfuzosin; baclofen; candesartan, clemastine, flunarizine, danazol, telmisartan, raloxifene, triamterene, guanfacine, disulfiram, and cinnarizine, respectively. (B) Increase in AcH3 in H1975 after incubation with cinnarizine (10 μM), triamterene (10 μM), or flunarizine (10 μM) for the indicated time (4, 12, or 24 h). SAHA (2 μM) served as the control for comparison. (C) Increase in histone H3 acetylation and α-tubulin acetylation in H1975 after incubation with different flunarizine (1.25, 2.5, 5, or 10 μM) or SAHA (0.25, 0.5, 1, or 2 μM; as control) concentrations for 24 h. Representative data from three independent experiments are shown. (D) Data from the cell-free biochemical HDAC activity assay demonstrating the inhibition of total HDAC activity from the H1975 nuclear extract by flunarizine or SAHA (control HDACI) in a concentration-dependent manner.

An increase of histone acetylation is a well-characterized hallmark of HDAC inhibition. The acetylation level of histone H3 (AcH3) was examined in H1975 cells after 24 h of incubation with the HDACI candidates at 10 μM (Figure 1A). The clinically approved drug SAHA (2 μM) was used as a positive control HDACI. Among the drug candidates evaluated, cinnarizine, flunarizine, and triamterene were shown to increase the level of histone H3 acetylation appreciably in H1975 cells, with flunarizine showing the most remarkable HDAC inhibition in H1975 cells (Figure 1A). The fold increase in histone H3 acetylation (AcH3) achieved by cinnarizine, flunarizine, and triamterene over the vehicle control was 15 ± 3, 89 ± 7, and 75 ± 6, respectively. Compared with the control HDACI (SAHA, 2 μM) which produced a remarkable increase in AcH3 after 4 h (520 ± 32-fold increase over the vehicle control), it required a longer drug incubation time (24 h) for flunarizine (10 μM) to notably increase AcH3 (89 ± 7-fold increase over the vehicle control after 24 h of treatment) in H1975 cells (Figure 1B). H1975 cells were also incubated with a range of different fluorinase concentrations (1.25, 2.5, 5, or 10 μM) or SAHA concentrations (0.25, 0.5, 1, or 2 μM) for 24 h. A concentration-dependent increase in AcH3 by flunarizine was observed, but it was less effective than SAHA (Figure 1C).

There are four major classes of HDAC enzymes, i.e., class I (HDAC 1, 2, 3, and 8), class II (HDAC 4–7, 9, 10), class III (SIRTs 1–7), and class IV (HDAC 11).^5^ Class I, II, and IV HDACs are known to reduce the post-translational acetylation of lysine residues in histones and various other cellular proteins. On the other hand, class III HDACs are specific NAD-dependent deacetylases, which can be inhibited by nicotinamide. In this study, apart from acetylated histone H3 (class I HDAC substrate), the protein acetylation level of a representative class II HDAC substrate (α-tubulin) was also examined in NSCLC cells after incubation with the HDACI candidates. In agreement with the known inhibition of both class I and II HDACs by SAHA, the level of acetylation of histone H3 and α-tubulin was increased by SAHA in a concentration-dependent manner (Figure 1C). At the highest concentration tested, SAHA (2 μM) increased α-tubulin acetylation by 105 ± 18-fold and histone H3 acetylation by 636 ± 42-fold over the vehicle control after 24 h of treatment. On the contrary, flunarizine (10 μM, the highest concentration tested) was found to increase AcH3 only (by 78 ± 6-fold over the vehicle control), but it did not affect acetylation of α-tubulin (Figure 1C).

The HDAC inhibitory effect of flunarizine was also examined by a cell-free biochemical HDAC activity assay. The inhibition of total HDAC activity by flunarizine (0.3125–10 μM) in the nuclear extract isolated from H1975 cells was tested. Flunarizine (at ≥0.625 μM) was found to show concentration-dependent HDAC inhibition, but this effect was weaker than SAHA (control HDACI) (Figure 1D). At the highest concentration tested, SAHA (10 μM) and flunarizine (10 μM) were shown to inhibit ∼90 and ∼75% of total HDAC activity from the H1975 nuclear extract, respectively. Furthermore, the HDAC isoform selectivity of flunarizine was investigated by using individual recombinant HDAC proteins (HDAC1, 2, 3, 6, and 8) in the biochemical HDAC activity assay. As summarized in Table 2, SAHA exhibited a similar inhibitory effect on class I (HDAC1–3) and class II (HDAC6) (IC_50_ in the range of 48–82 nM). In contrast, flunarizine was more selective in inhibiting class I (HDAC1–3) (IC_50_ in the range of 285–360 nM) than class II (HDAC6) (IC_50_ ∼ 5600 nM). Both SAHA and flunarizine displayed a lower inhibitory effect against HDAC8 (IC_50_ = 620 ± 24 and 5500 ± 450 nM, respectively) than other HDAC isoforms.

**Table 2:** Inhibition of Recombinant HDAC1, 2, 3, 6, and 8 Isoforms by SAHA and Flunarizine^a^

	IC_50_ (nM)				
compound	HDAC1	HDAC2	HDAC3	HDAC6	HDAC8
SAHA	55 ± 3	82 ± 9	48 ± 6	52 ± 4	620 ± 24
flunarizine	285 ± 15	360 ± 26	305 ± 31	5600 ± 600	5500 ± 450

a IC_50_ values for SAHA and flunarizine from a biochemical HDAC activity assay using the indicated recombinant HDAC isoforms were reported as mean ± standard deviation (three independent experiments).

### Flunarizine Potentiated the Anticancer Proliferative Effect of Gefitinib More Significantly in Gefitinib-Resistant NSCLC Cell Lines

The circumvention of gefitinib resistance was investigated in a few NSCLC cell lines harboring different resistance mechanisms (H1975, A549, H1650, H820; gefitinib-sensitive HCC827 cell line was used as a control). As a control for comparison, the combination of equipotent concentrations of gefitinib and SAHA (control HDACI) was evaluated by median effect analysis. Different degrees of synergism were observed for gefitinib–SAHA combination in the cell lines (combination index (CI) < 1 suggests a synergistic anticancer effect) (Table 3A). More remarkable synergism was observed in the gefitinib-resistant cell lines (CI = 0.36 ± 0.07, 0.58 ± 0.11, 0.64 ± 0.09, and 0.72 ± 0.06 in H1975, A549, H1650, and H820, respectively) than in the gefitinib-sensitive HCC827 cell line (CI = 0.88 ± 0.10) upon treatment with the gefitinib–SAHA combination.

**Table 3:** Flunarizine Sensitized Gefitinib-Resistant NSCLC to Gefitinib to Different Extents

(A) Evaluation by Combination Index (CI) Method for the Control HDACI (SAHA) Which Exhibited Remarkable Anticancer Activity on Its Own. CI < 1 Suggests Synergistic Cytotoxicity					
	sensitive cells	resistant cells			
CI	HCC827	H1975	A549	H1650	H820
SAHA	0.88 ± 0.10	0.36 ± 0.07	0.58 ± 0.11	0.64 ± 0.09	0.72 ± 0.06

(B) Evaluation by the Change in % Cell Viability for the Selected HDACI Candidate (Flunarizine) As It Exhibited Only a Mild Anticancer Effect Alone (<20% Reduction in Cell Viability at the Highest Concentration Tested)				
cell viability (%)	[flunarizine] μM			
	0	2.5	5	10
H1975 Cells				
no gefitinib	100%	95 ± 8%	94 ± 6%	86 ± 7%
+7.5 μM gefitinib	61 ± 5%	52 ± 4%	38 ± 4%^a^	21 ± 6%^a^
A549 Cells				
no gefitinib	100%	101 ± 5%	95 ± 6%	102 ± 8%
+7.5 μM gefitinib	59 ± 7%	52 ± 3%	47 ± 3%	48 ± 5%
H1650 Cells				
no gefitinib	100%	93 ± 4%	92 ± 5%	87 ± 6%
+7.5 μM gefitinib	52 ± 8%	56 ± 3%	47 ± 4%	42 ± 7%
H820 Cells				
no gefitinib	100%	103 ± 5%	94 ± 7%	89 ± 5%
+7.5 μM gefitinib	66 ± 8%	68 ± 5%	62 ± 5%	57 ± 4%

(C) Different Dosing Schedules of Flunarizine (10 μM) and Gefitinib (7.5 μM) Combination Gave Similar Potentiation in the Anticancer Effect in H1975 Cells			
cell viability (%)	simultaneous	flunarizine-preceding-gefitinib	gefitinib-preceding-flunarizine
gefitinib + flunarizine	21 ± 6%	26 ± 3%	23 ± 5%

a *p* < 0.05, compared with gefitinib treatment alone without flunarizine.

As flunarizine produced the most remarkable increase in AcH3 among the positive HDAC-inhibiting drug candidates, it was selected for detailed study in gefitinib-resistant NSCLC cell lines with different resistance mechanisms (H1975-EGFR T790M; A549-KRAS G12S; H1650-PTEN loss; H820-EGFR T790M; and *MET* amplification) (Table 3B). Since flunarizine alone did not significantly affect cancer cell viability by more than 25% even at the highest concentration tested (20 μM), the combination index method was not used to evaluate the gefitinib–flunarizine combination. Instead, the enhancement of the anticancer effect of gefitinib by a fixed flunarizine concentration was investigated. As summarized in Table 3B, flunarizine (2.5, 5, or 10 μM) was shown to potentiate the anticancer effect of gefitinib in all NSCLC cell lines tested. However, more remarkable potentiation was observed in H1975 cells (bearing the EGFR T790 M mutation). In gefitinib–flunarizine combination-treated H1975 cells, simultaneous drug exposure gave similar potentiation of the anticancer effect as the dosing schedules with flunarizine preceding gefitinib or vice versa (Table 3C).

### Flunarizine Promoted Gefitinib-Mediated Apoptosis More Significantly in Resistant Cells

**Figure 2:**
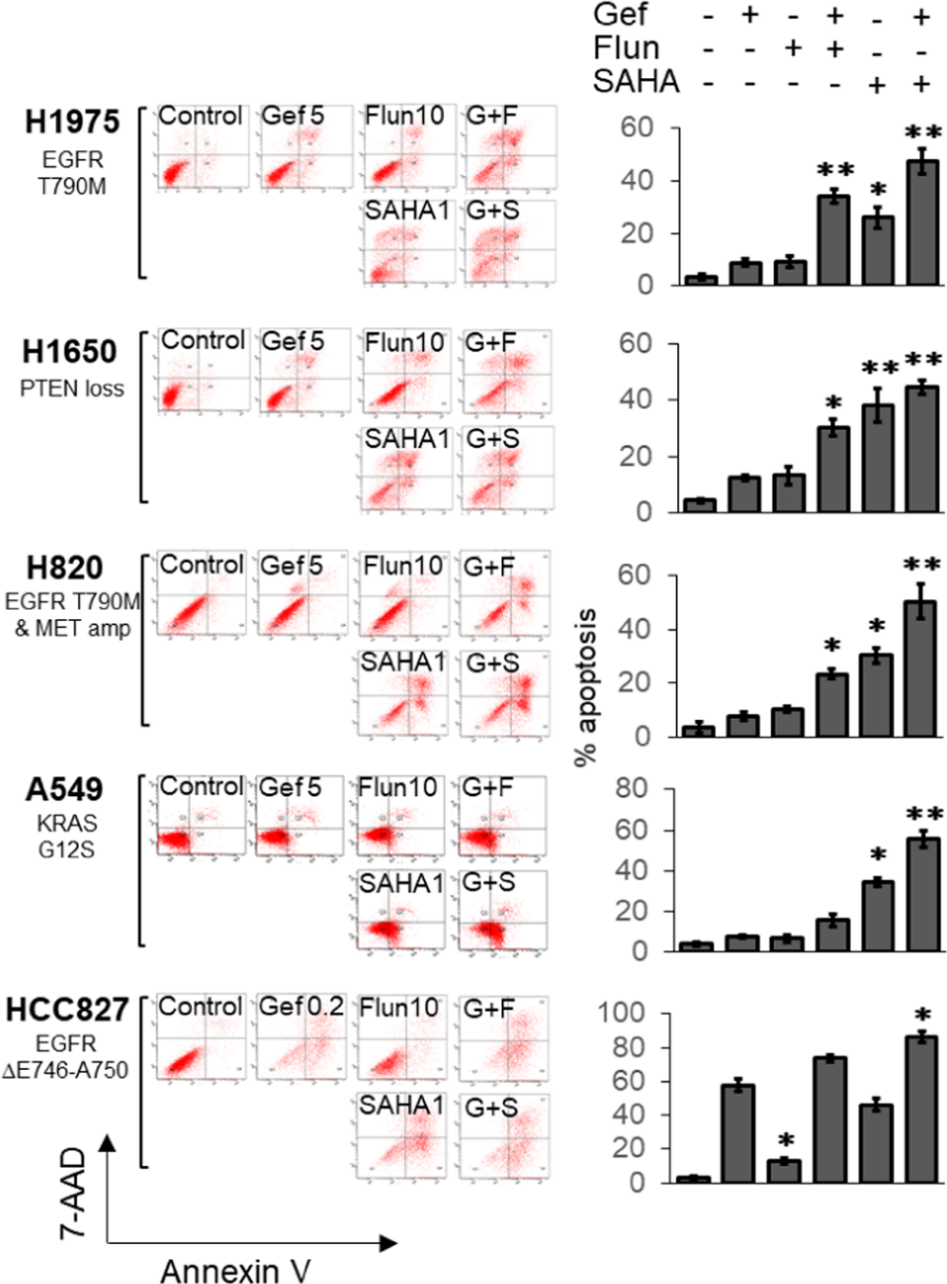
Flunarizine significantly potentiated gefitinib-induced apoptosis in the resistant NSCLC cell lines. Gefitinib-resistant cell line (H1975, H1650, H820, and A549) and gefitinib-sensitive cell line (HCC827) were treated with gefitinib (Gef), flunarizine (Flun), SAHA alone, or other combination (G + F = Gef + Flun; G + S = Gef + SAHA) at the indicated concentration (in μM) for 48 h. Apoptosis was measured by the standard flow cytometry-based Annexin V/7-AAD assay. (Left panel) Representative dot plots from three independent experiments showing apoptosis induction. (Right panel) The apoptotic cell population (% of total cell population) is presented as mean ± standard deviation (SD) *p < 0.05, **p < 0.01; compared with gefitinib treatment alone.

In our study, gefitinib was shown to produce remarkably less apoptosis in the four gefitinib-resistant NSCLC cell lines (H1975, H1650, A549, and H820) than in the sensitive HCC827 cells (∼5–10% upon 5 μM gefitinib treatment in H1975, H1650, A549, and H820 vs ∼60% apoptosis after 0.2 μM gefitinib treatment in HCC827) (Figure 2). The increase in apoptosis by flunarizine (10 μM) or the control HDACI SAHA (1 μM) alone was also examined after 48 h of treatment. SAHA was found to trigger a more significant apoptotic effect than flunarizine in all NSCLC cell lines tested (Figure 2). Importantly, flunarizine (10 μM) could remarkably potentiate gefitinib (5 μM)-induced apoptosis, from 8.9 ± 1.3, 12.4 ± 0.9, 7.6 ± 0.9, and 7.9 ± 1.6% (gefitinib alone) to 34.1 ± 2.7, 30.1 ± 2.9, 15.7 ± 2.9, and 23.5 ± 1.7% (gefitinib–flunarizine combination) in H1975, H1650, A549, and H820, respectively (Figure 2). On the other hand, the enhancement of gefitinib-mediated apoptosis by flunarizine in the sensitive HCC827 cells was significantly less impressive (Figure 2). The cancer cell population in apoptosis was only increased from 67.8 ± 3.9% (gefitinib 0.2 μM alone) to 73.7 ± 2.2% (gefitinib–flunarizine combination) (Figure 2).

### Gefitinib–Flunarizine Combination Led to More Significant G1 Cell Cycle Arrest Than the Two Individual Drugs Alone

**Figure 3:**
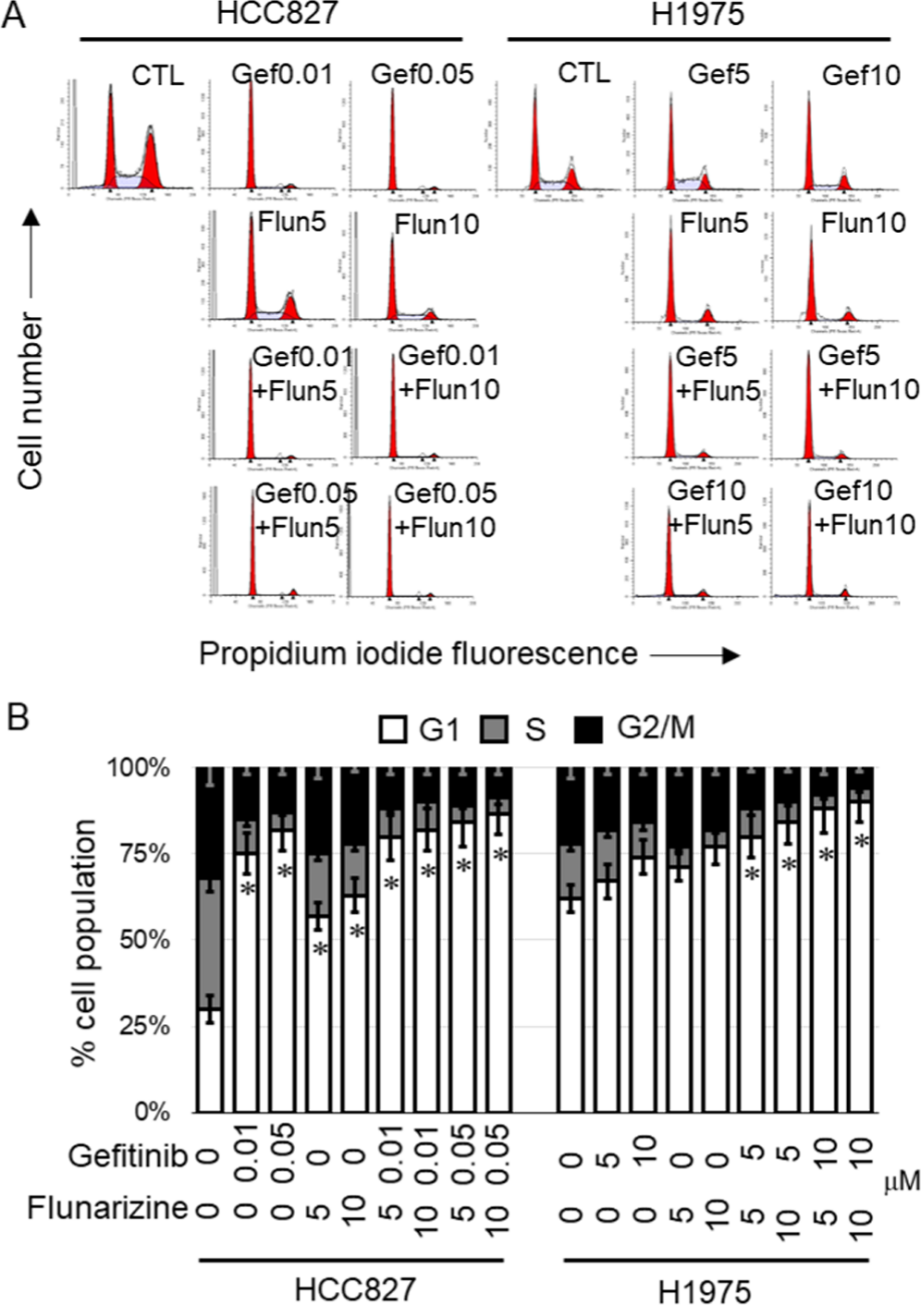
Flunarizine (Flun) promoted gefitinib (Gef)-induced G1 cell cycle arrest more significantly in gefitinib-resistant H1975 cells. (A) Profile of cell cycle progression in HCC827 (gefitinib-sensitive) or H1975 (gefitinib-resistant) cells after 24 h of treatment with gefitinib (0.01, 0.05, 5, or 10 μM) alone in the presence or absence of flunarizine (5 or 10 μM) evaluated by the standard flow cytometry-based propidium iodide staining method. (B) Representative data from three independent experiments are shown as mean ± SD *p < 0.05, compared with the G1 phase in no treatment control.

EGFR-TKIs are known to retard cell cycle progression to elicit their anticancer effect.^13,14^ HCC827 and H1975, representing gefitinib-sensitive and -resistant cells, respectively, were incubated with flunarizine (5 or 10 μM) alone or its combination with gefitinib (0.01/0.05 μM in HCC827 or 5/10 μM in H1975 cells) for 24 h. Consistent with literature-reported findings, gefitinib produced much less G1 arrest in H1975 (gefitinib-resistant) than in HCC827 (gefitinib-sensitive) cells (Figure 3). Thus, the less effective G1 cell cycle arrest following gefitinib treatment in our cell models is associated with resistance to the TKI. Interestingly, the HDACI candidate flunarizine alone was shown to induce G1 cell cycle arrest (Figure 3). More importantly, the flunarizine–gefitinib combination produced more remarkable G1 arrest than the individual drugs alone in the gefitinib-resistant H1975 cells, presumably contributing to circumvention of gefitinib resistance (Figure 3).

### Flunarizine Upregulated Bim and p27^Kip1^ by Increasing Histone Acetylation in the Gene Promoters

**Figure 4:**
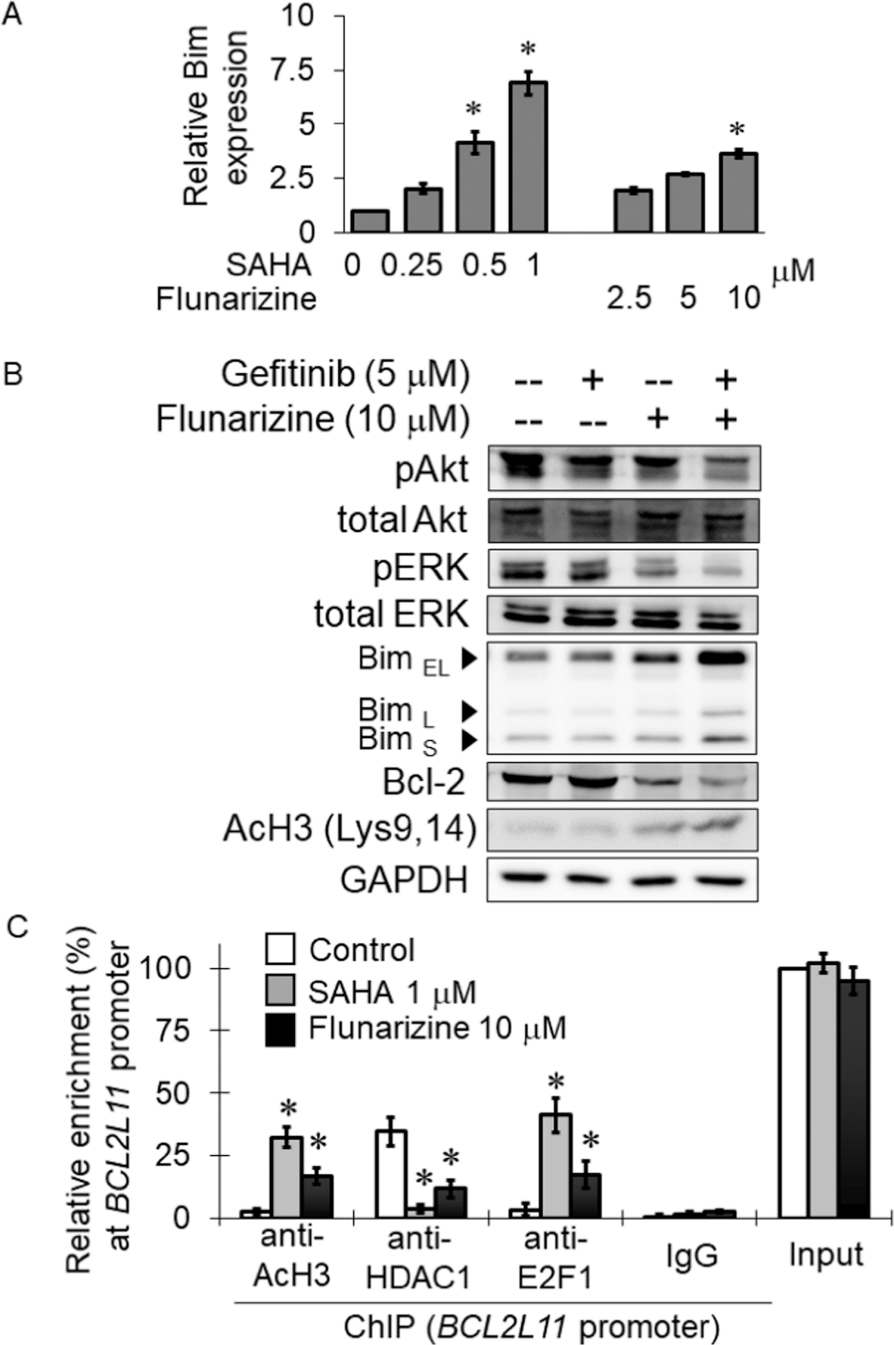
Flunarizine upregulated the Bim expression to induce apoptosis in H1975 cells. (A) Real-time polymerase chain reaction (PCR) data showing the concentration-dependent increase in the Bim expression by flunarizine. SAHA was used as a positive control. The house-keeping gene GAPDH was used as the loading control for normalization. Representative data from three independent experiments are shown as mean ± SD *p < 0.05; compared with the no-treatment control. (B) Western blot analysis data showing the upregulation of the pro-apoptotic Bim but downregulation of the antiapoptotic Bcl-2 protein by gefitinib (5 μM)–flunarizine (10 μM) combination preferentially in H1975 cells. While H1975 cells are resistant to gefitinib and did not exhibit appreciable inhibition of pAkt and pERK upon gefitinib treatment, the gefitinib–flunarizine combination gave rise to significantly more remarkable inhibition of pAkt and pERK than gefitinib alone. (C) Flunarizine upregulated BIM in H1975 cells by decreasing the binding of HDAC1 but increasing histone H3 acetylation and E2F1 enrichment on the Bim (*BCL2L11*) promoter after 24 h of incubation with flunarizine (10 μM) or SAHA (1 μM). The results are expressed as relative enrichment of the promoter (percentage of immunoprecipitate over total input DNA). Mean ± SD from three independent experiments is shown. *p < 0.05, compared with no treatment control.

**Figure 5:**
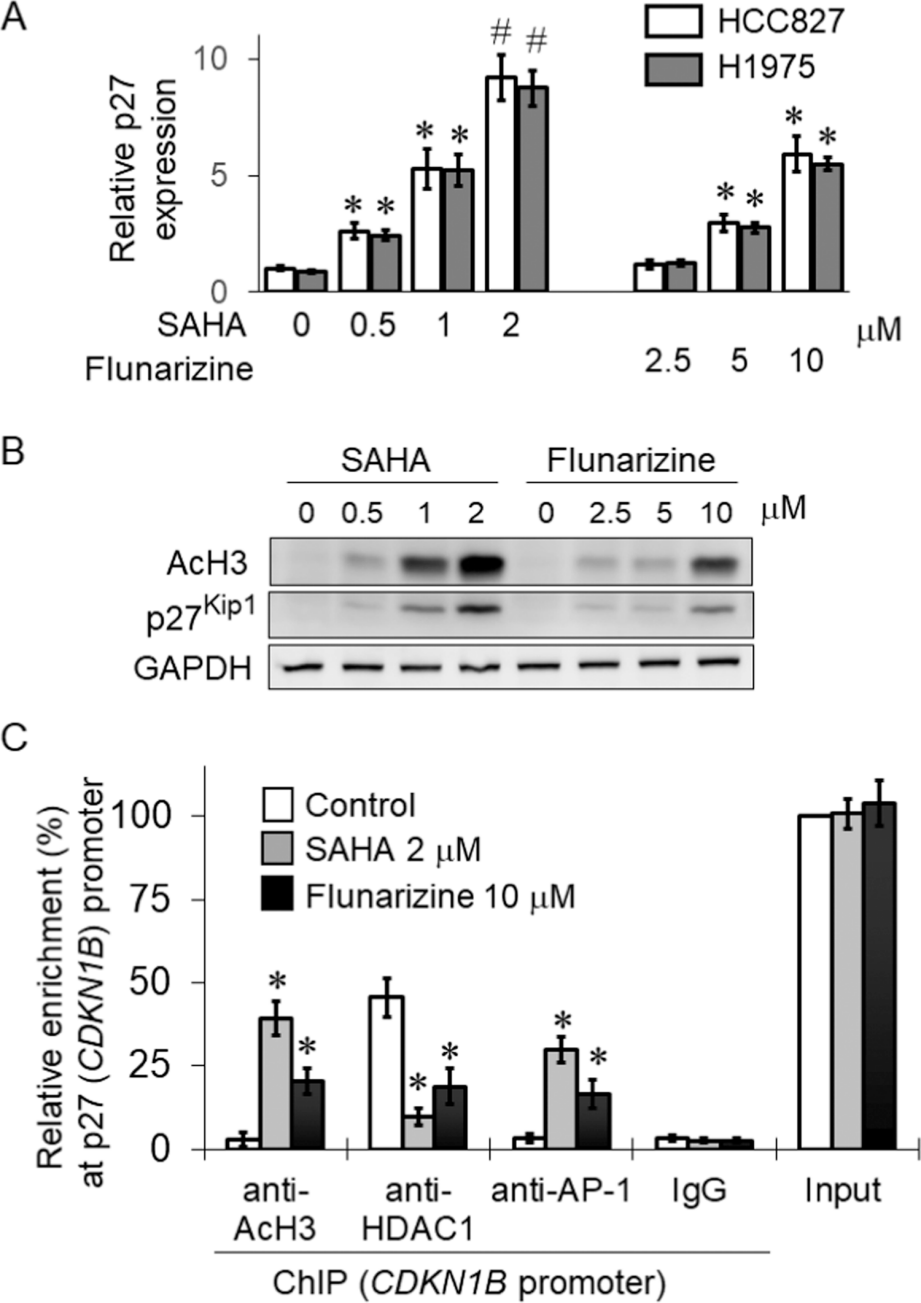
Flunarizine upregulated the p27^Kip1^ expression to mediate cell cycle arrest in HCC827 and H1975 cells. (A) Real-time PCR data showing the concentration-dependent upregulation of p27^Kip1^ by flunarizine. SAHA was used as a positive control. The house-keeping gene GAPDH was measured for normalization. Representative data from three independent experiments are shown as mean ± SD *p < 0.05; compared with no treatment control. (B) Western blot data showing the concentration-dependent increase of protein expression of p27^Kip1^ by SAHA and flunarizine in H1975 cells, which paralleled the induction of histone acetylation (AcH3). (C) Flunarizine induced p27^Kip1^ expression in H1975 cells by reducing the interaction of HDAC1 but increasing histone H3 acetylation and AP-1 enrichment at p27^Kip1^ (*CDKN1B*) promoter after 24 h of incubation with flunarizine (10 μM) or SAHA (2 μM). The results are expressed as relative enrichment of the promoter (percentage of immunoprecipitate over total input DNA). Mean ± SD from three independent experiments is shown. *p < 0.05, compared with no treatment control.

Bim is an apoptotic protein, encoded by the *BCL2L11* gene, which plays a critical role in mediating EGFR-TKI-induced apoptosis.^15^ p27^Kip1^ is known to regulate the G1 cell cycle arrest after gefitinib treatment.^16^ The expression of Bim and p27^Kip1^ was measured in H1975 following 24 h of incubation with flunarizine (2.5, 5, or 10 μM) or SAHA (0.25, 0.5, or 1 μM). Importantly, at all concentrations tested, both flunarizine and SAHA were found to significantly increase Bim (Figure 4A) and p27^Kip1^ (Figure 5A) concentrations in a concentration-dependent manner. Moreover, in H1975 cells, the flunarizine–gefitinib combination was shown to remarkably increase the expression of Bim (Figure 4B) and p27^Kip1^ (Figure 5B) but reduce the expression of the antiapoptotic protein Bcl-2 (Figure 4B), presumably circumventing gefitinib resistance.

A hallmark feature of HDACIs is that they can open up the chromatin at gene promoters, thereby inducing gene activation. ChIP assay was carried out to assess the binding of HDAC1 and two representative transcription factors (E2F1 and c-JUN) to the gene promoter of Bim (*BCL2L11*) and p27Kip1 (*CDKN1B*), respectively. Histone acetylation (AcH3) at the two gene promoters was also measured. Specific antibodies against AcH3, HDAC1, E2F1, or c-JUN were used to pull down the respective nuclear proteins in chromatin harvested from H1975 cells after SAHA (1 μM) or flunarizine (10 μM) treatment. The relative enrichment of these nuclear proteins on the *BCL2L11* or *CDKN1B* promoter was measured by quantitative real-time PCR. When the chromatin samples were pulled down by the IgG isotype control antibody, no PCR signal was obtained. Therefore, the immunoprecipitation reactions were specific (Figures 4C and 5C). A similar trend was observed after SAHA and flunarizine treatment, albeit a more remarkable effect was observed after SAHA treatment (Figures 4C and 5C). After SAHA or flunarizine incubation, significantly less binding of HDAC1 with the promoters of *BCL2L11* and *CDKN1B* and a remarkable increase in AcH3 enrichment were observed, thus suggesting a more relaxed chromatin configuration. There was also a remarkable increased binding of the transcription factor E2F1 and c-JUN to *BCL2L11* and *CDKN1B* promoters, respectively, thereby upregulating the two genes after SAHA or flunarizine treatment (Figures 4C and 5C).

### Flunarizine Overcame Gefitinib Resistance by Reversing Epithelial-Mesenchymal Transition (EMT)

**Figure 6:**
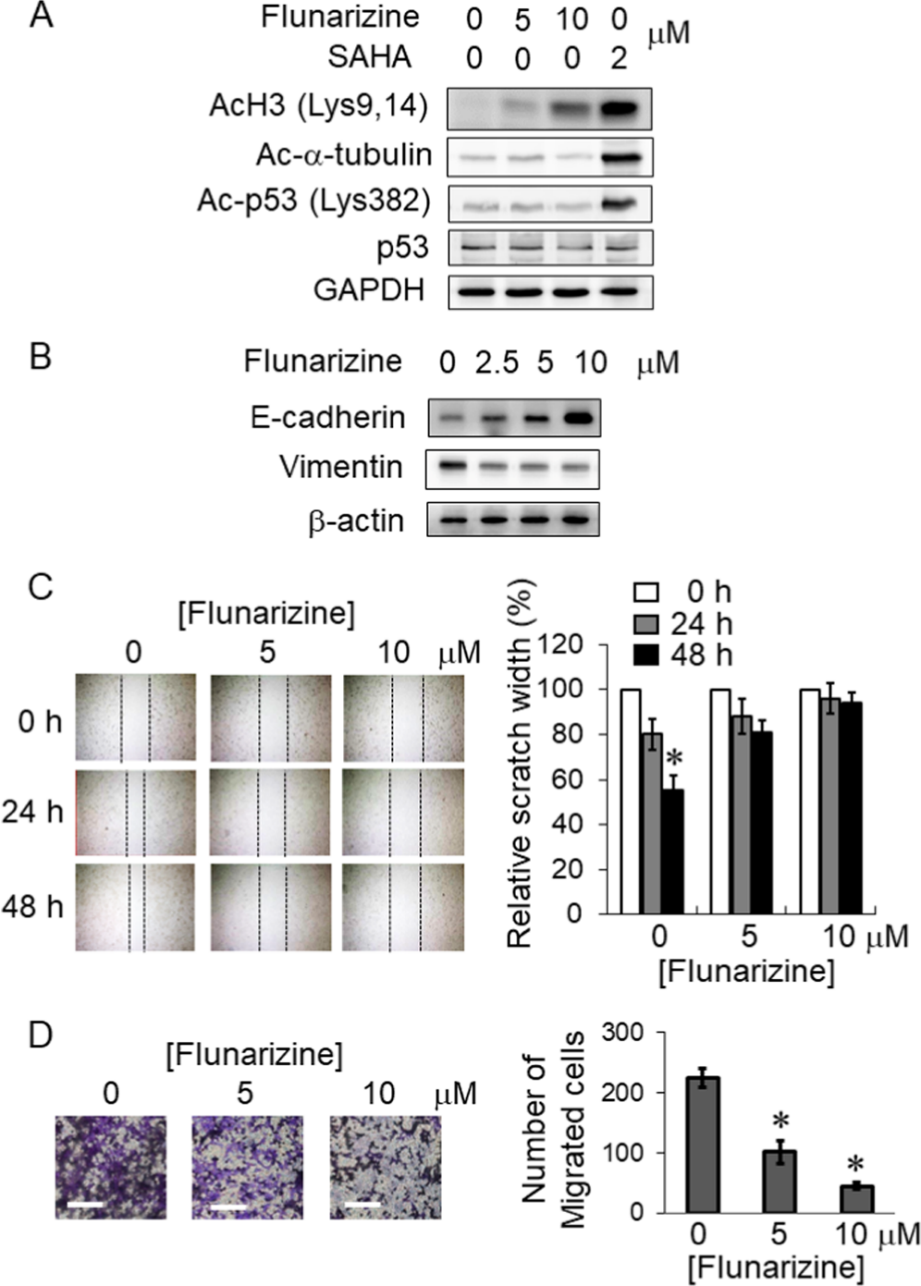
Flunarizine also overcame gefitinib resistance by reversing epithelial-mesenchymal transition (EMT). (A) Western blot analysis data showing that flunarizine at gefitinib resistance circumvention concentrations did not affect the acetylation status of α-tubulin and p53. SAHA was used as a control, which is known to promote acetylation of histone H3, α-tubulin, and p53. (B) Flunarizine increased E-cadherin but reduced the vimentin protein level in a concentration-dependent manner in H1975 cells. (C) Flunarizine inhibited the cell migration ability of H1975 cells in wound healing assay. (D) Transwell invasion assay. Representative images of migrated cells after staining with crystal violet were shown. Scale bar, 100 μm. The number of counted cells was reported. *p < 0.05 compared with the no treatment control.

HDACIs are known to regulate the post-translational acetylation of nonhistone proteins to produce a pleiotropic cellular effect.^17^ To this end, flunarizine (at a concentration sufficient to overcome gefitinib resistance and up to 10 μM) did not affect the acetylation of a few other representative nonhistone proteins (including α-tubulin and p53) (Figure 6A). On the other hand, epithelial-mesenchymal transition (EMT) plays a critical role in cancer invasion and metastasis and it is known to contribute to EGFR-TKI resistance.^18^ HDACIs have been reported to reverse EMT by upregulating E-cadherin.^19^ In H1975 cells, flunarizine was found to upregulate E-cadherin but downregulate vimentin expression in a concentration-dependent manner (Figure 6B), presumably leading to the inhibition of cancer cell migration (Figure 6C) and invasion (Figure 6D).

### Flunarizine Enhanced the Antitumor Activity of Gefitinib in an EGFR-TKI-Refractory PDX Model

**Figure 7:**
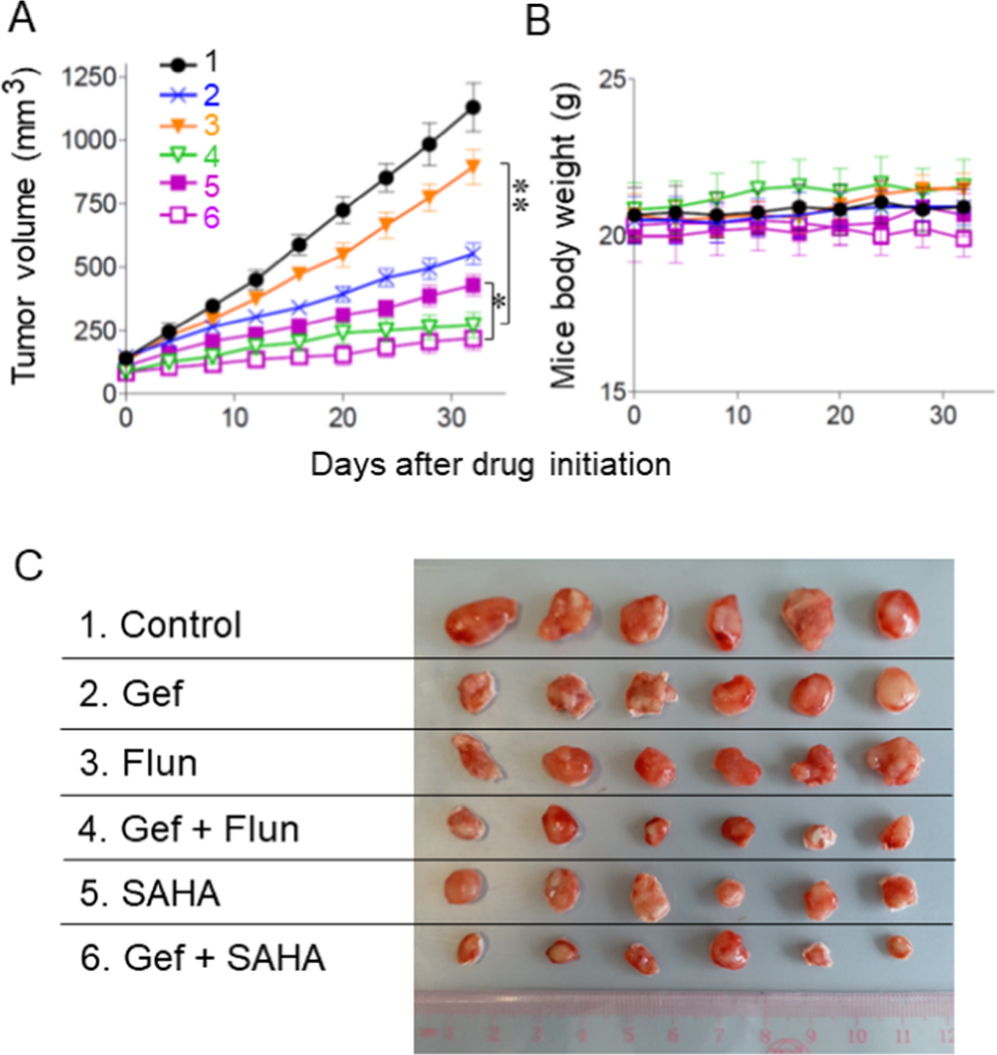
Gefitinib–flunarizine combination significantly suppressed tumor growth of erlotinib–refractory PDX from an Asian NSCLC patient than the individual drugs alone, suggesting its potentiation for circumventing gefitinib resistance in vivo. (A) Tumor growth progression during the drug treatment. 1: Vehicle control; 2: gefitinib alone; 3: flunarizine alone; 4: gefitinib + flunarizine combination; 5: SAHA alone; and 6: gefitinib + SAHA combination. *n* = 6 in each treatment group. *p < 0.05; SAHA alone vs gefitinib + SAHA combination. **p < 0.01; flunarizine alone vs gefitinib + flunarizine. (B) The various treatment groups did not significantly affect mice body weight. (C) Tumor xenografts were excised at the end of the study (day 32).

The antitumor efficacy of gefitinib alone, flunarizine alone, SAHA alone, or combinations of gefitinib and flunarizine/SAHA was evaluated in a PDX model from an Asian NSCLC patient presenting disease progression after erlotinib treatment. The tumor specimen was found to carry EGFR mutations L858R and T790 M. Compared with the control mice at termination of the experiment (day 32), tumor volume was found to be 535 ± 124, 834 ± 143, 285 ± 68, 368 ± 101, and 226 ± 33 mm^2^ for gefitinib alone, flunarizine alone, gefitinib + flunarizine combination, SAHA alone, and gefitinib + SAHA combination, respectively (vs 1108 ± 223 mm^2^ for the control mice). The gefitinib + flunarizine combination group was found to produce more significant tumor growth than the gefitinib-alone group (*p* < 0.01) (Figure 7A,C). It is noteworthy that the gefitinib–flunarizine combination exhibited only a slightly lesser antitumor effect than that of the gefitinib–SAHA combination, and the difference was not statistically significant. The drug combination did not cause significant animal body weight loss or animal death (Figure 7B).

### Flunarizine Did Not Affect the Major ABC Efflux Transporters and Tumoral Accumulation of Gefitinib

**Figure 8:**
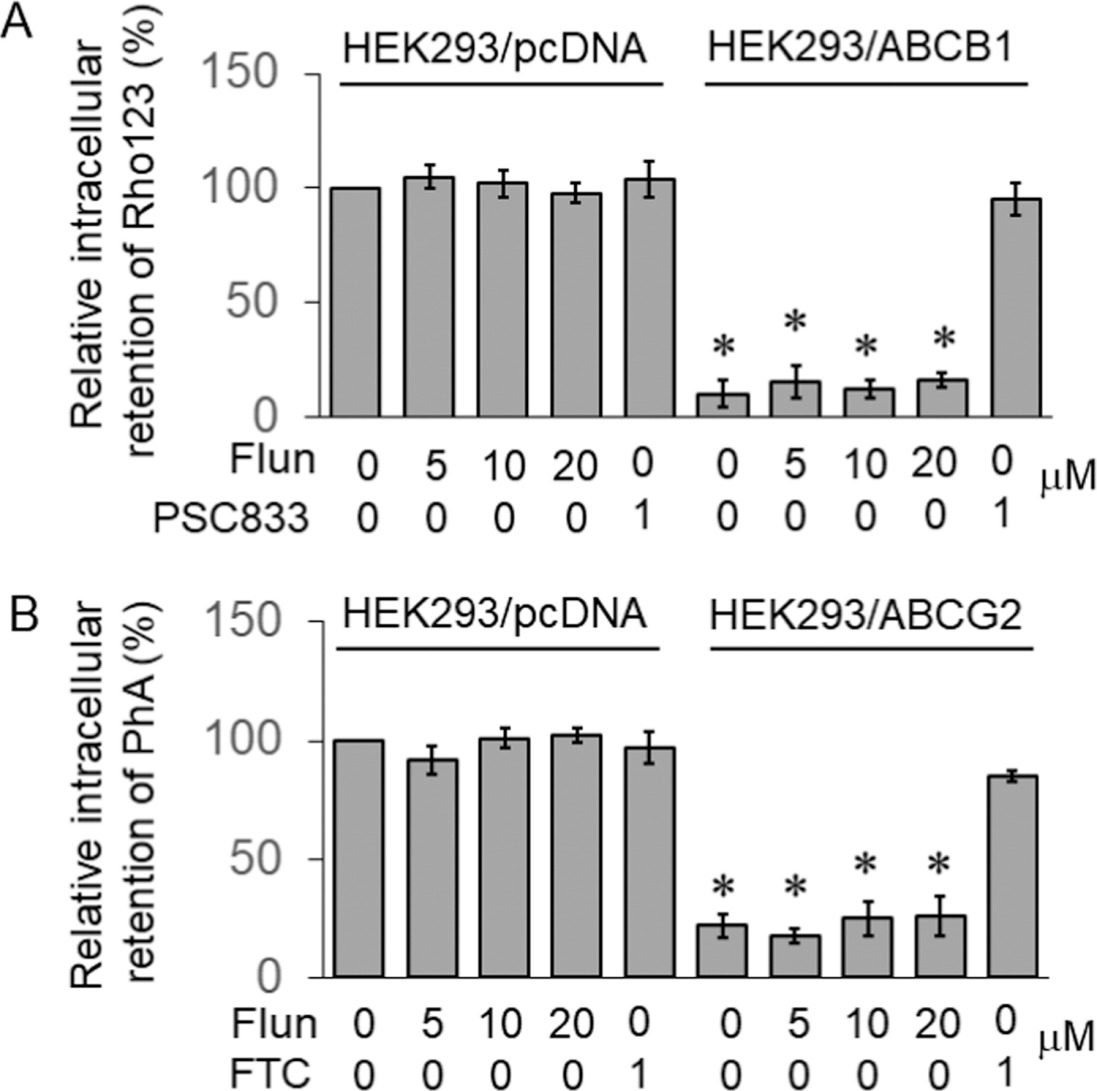
Effect of flunarizine on the cellular efflux of fluorescent ABCB1 (rhodamine 123 (Rh123)) and ABCG2 (pheophorbide A (PhA)) substrates in stably transfected HEK293/pcDNA3 (vector control), HEK293/ABCB1 (ABCB1-overexpressing), or HEK293/ABCG2 (ABCG2-overexpressing) cells. The retention of Rh123 (A) or PhA (B) after a 1 h drug-free efflux period was measured by flow cytometry. The results are presented as the percentage fluorescence intensity relative to HEK293/pcDNA3 (control) cells. Data are reported as mean ± standard deviation from three independent experiments.

A few drug efflux transporters (including ABCB1 and ABCG2) have been reported to mediate gefitinib resistance.^20^ The possible ABCB1 and ABCG2 transporter inhibitory effect of flunarizine was evaluated by a flow cytometric assay to measure the efflux of fluorescent ABCB1 or ABCG2 probe substrates (rhodamine 123 and pheophorbide A, respectively). While the control ABCB1 (PSC833, 1 μM) and ABCG2 (FTC, 1 μM) inhibitors potently inhibited the efflux of rhodamine 123 and pheophorbide A, flunarizine (up to 20 μM) did not affect the efflux of the fluorescent transporter substrates (Figure 8A,B). To investigate the possible effect of flunarizine on bioavailability and tumor accumulation of gefitinib in vivo, blood was drawn before animal sacrifice at 4 h after the last gefitinib dosing (approximately *t*_max_ of gefitinib) in the PDX study for the measurement of plasma gefitinib concentration by liquid chromatography with tandem mass spectrometry (LC-MS/MS). Tumor xenografts were also excised for determination of tumoral accumulation of gefitinib in the different treatment groups. Compared with gefitinib treatment alone, both flunarizine and SAHA (control HDACI) did not significantly affect the plasma concentration or tumoral accumulation of gefitinib (Table 5).

**Table 4:** Characteristic of the Patient-Derived Tumor Xenograft (PDX) Model Used in the Study^a^

PDX from an advanced NSCLC patient (TKI-resistant)	
patient race/ethnicity	Chinese
tumor characteristic	(i) lung cancer with mediastinal/SCF LNs, pleural effusion, and pericardial effusion
	(ii) adenocarcinoma (TTF-1+ve/calretinin–ve) with L858R mutation+ve/ALK–ve (pleural fluid); gum biopsy: NSCLC with similar morphology but TTF-1–ve
	(iii) Sanger sequencing of genomic DNA obtained from the PDX tumor confirmed the presence of EGFR L858R and T790M
treatment history	(i) erlotinib for 5 months, then progressive disease
	(ii) pleural fluid (on 5th month after erlotinib therapy) – EGFR L858R and T790 M mutation +ve, but lung biopsy EGFR L858R+/T790M–
	(iii) pemetrexed-carboplatin wk 1/erlotinib wk (2–3) × 3 cycles (for the following 3 months)
	(iv) PET-CT (after 3 cycles of pemetrexed-carboplatin/erlotinib): progressive disease, multiple new lung metastases, and new bone metastases
	(v) osimertinib (for the following 3 months)
	(vi) patient succumbed after 3 month osimertinib therapy

a Abbreviations: ALK = ALK receptor tyrosine kinase; PEP-CT = positron emission tomography and computerized tomography scan; SCF LNs = supraclavicular lymph nodes; TKI = tyrosine kinase inhibitor; and TTF-1 = thyroid transcription factor 1.

## Discussion

In recent years, there has been an escalating research interest in adopting the drug repurposing approach to enhance the efficacy of cancer therapy. Numerous data mining and biological network analytical methods have been used to identify drug-repurposing candidates using different biomedical databases.^21,22^ In this study, we used an open-source web-based program called “DRUGSURV” to identify HDACI candidates from clinically approved non-oncology drugs. The data mining principle of DRUGSURV relies on the drug signature and disease signature compiled in its database.^23^ “Drug signature” is defined by the modulation of molecular targets/genes/proteins after drug treatment, whereas “disease signature” refers to the alterations of genes in a particular disease condition. A significant negative correlation between the drug signature and disease signature may suggest possible application of the drug to treat the particular disease.^24^ Besides, two drugs showing similar drug signatures may be used for similar therapeutic indications. It is noteworthy that DRUGSURV uses genes significantly associated with patient survival as a disease signature. Therefore, repurposing drug candidates identified by DRUGSURV may prolong patient survival by modulating the gene(s) used during the query process.^23^

In the present study, the circumvention of gefitinib resistance by the newly identified HDACI candidate (flunarizine) was tested in human NSCLC cell lines displaying a range of key gefitinib resistance mechanisms (secondary EGFR T790 M (in H1975), KRAS mutation (in A549), PTEN loss (in H1650), and *MET* amplification (in H820)). Flunarizine is a selective calcium channel blocker with a histamine H1 blocking activity. It is clinically approved for the prophylaxis of migraine and treatment of vertigo.^25,26^ Interestingly, flunarizine has been recently shown to exhibit in vitro cytotoxic activity in glioblastoma multiforme and multiple myeloma.^27,28^ However, the underlying mechanism was not elucidated. Moreover, the novel biological function of flunarizine as an HDACI has not been reported. In addition, the combination of flunarizine and anticancer drugs with an aim to overcome drug resistance has not been systematically investigated.

The HDAC inhibitory effect of flunarizine was confirmed by the cell-free biochemical HDAC activity assay (Figure 1). At the highest concentration tested, flunarizine exhibited less potent inhibition of total HDAC activity than SAHA (∼75 vs ∼90% inhibition) in the nuclear extract of H1975 NSCLC cells (Figure 1C).^29−31^ Current research efforts focused on developing selective HDACIs with an aim to reduce toxicity due to off-target effects and nonspecific inhibition of HDACs by pan-HDACIs. To this end, flunarizine was found to be more selective toward class I HDACs (HDAC1, 2, and 3) than class II HDAC (HDAC6) (Table 2), which is distinctly different from the pan-HDAC inhibitor (SAHA). The HDAC isoform selectivity of flunarizine was also reflected by its differential effect on the acetylation of histone H3 (substrate of class I HDACs) vs α-tubulin (substrate of HDAC6) (Figure 1C). At the highest concentration tested, flunarizine (10 μM) was showed to increase AcH3 only, but it did not affect acetylation of α-tubulin.

While flunarizine alone produced only a modest anticancer effect in the NSCLC cell lines tested, it was shown to remarkably enhance the anticancer effect of gefitinib in all gefitinib-resistant NSCLC cell lines tested in a concentration-dependent manner (Table 3). BEAS-2B is a normal human bronchial epithelial cell line widely used for toxicity evaluation of xenobiotics to lung tissues in vitro.^32^ Flunarizine alone did not appreciably affect the cell viability of BEAS-2B. Importantly, the gefitinib–flunarizine combination did not exacerbate the toxicity compared with gefitinib treatment alone in BEASE-2B cells (Supp. Figure 1). Therefore, the gefitinib–flunarizine combination may exhibit preferential cytotoxicity on cancer cells. More importantly, flunarizine was further shown to potentiate the antitumor activity of gefitinib in a PDX model derived from an Asian NSCLC patient refractory to erlotinib therapy, thus substantiating its capability to overcome gefitinib resistance *in vivo* (Figure 7). While flunarizine alone was shown to exhibit a less HDAC inhibitory effect (Figure 1) and antitumor activity (Figures 2 and 7) than SAHA alone, it is noteworthy that the gefitinib–flunarizine combination only displayed a slightly less antitumor effect than the gefitinib–SAHA combination in the PDX animal study and the difference was not statistically significant (Figure 7). To this end, the elimination half-life of flunarizine (around 18 days)^33^ was reported to be markedly longer than that of SAHA (∼2 h).^34^ This may partially explain the comparable antitumor effect of their combination with gefitinib in the PDX model.

The apoptotic and cell cycle inhibitory effects of flunarizine were further investigated. Flunarizine induced mild but concentration-dependent apoptosis in the NSCLC cell lines tested (Figure 2). Besides, the cell cycle arrest effect of flunarizine was also tested. Similar to the control HDACI (SAHA), flunarizine was shown to arrest cell cycle progression at the G1 phase in NSCLC cell lines (Figure 3). More importantly, the gefitinib–flunarizine combination was found to induce a more significant G1 arrest than the two individual agents alone (Figure 3). Since reduced apoptosis and cell cycle arrest are the major contributors to gefitinib resistance,^4^ these mechanisms may contribute to the circumvention of gefitinib resistance by the flunarizine–gefitinib combination. Our results are also in good agreement with previous publications about the promotion of apoptosis^35,36^ and induction of cell cycle arrest^37^ by other gefitinib/HDACI combinations in different cancer types.

The underlying mechanisms contributing to the induction of apoptosis and cell cycle arrest by flunarizine were further investigated. Gefitinib is known to upregulate Bim (*BCL2L11*)^38^ and p27^Kip1^ (*CDKN1B*)^39^ to elicit its cytotoxic effect and G1 cell cycle arrest, respectively. To this end, the mRNA expression of Bim (*BCL2L11*) and p27^Kip1^ (*CKDNB*) was elevated by flunarizine in a concentration-dependent manner (Figures 4 and 5). Therefore, the increase in Bim and p27^Kip1^ expression is likely responsible for the potentiated apoptotic effect and cell cycle arrest of the gefitinib–flunarizine combination.

To further understand the upregulation of Bim and p27^Kip1^ by flunarizine, the ChIP assay was performed to evaluate the binding of selected transcriptional factors to gene promoters following flunarizine treatment. Consistent with HDAC inhibition by flunarizine, it reduced the enrichment of HDAC1 and hence increased the level of histone acetylation on Bim (*BCL2L11*) (Figure 4C) and p27^Kip1^ (*CDKN1B*) promoters (Figure 5C). The result demonstrated a more relaxed chromatin configuration at the gene promoters after flunarizine treatment. Moreover, this was also accompanied by a stronger interaction of E2F1 with *BCL2L11* and c-JUN with *CDKN1B* gene promoters to induce *BCL2L11* and *CDKN1B* genes, respectively, after flunarizine treatment. To this end, E2F1 and c-JUN have been reported to regulate the transcription of Bim and p27^Kip1^,^40,41^ respectively. To the best of our knowledge, this study is the first detailed study about the dynamic modification of the chromatin architecture by flunarizine on its target genes.

As the major drug efflux transporters (including ABCB1 and ABCG2) are implicated in gefitinib resistance, the possible inhibition of ABCB1 and ABCG2 by flunarizine was also investigated. Our findings revealed that flunarizine did not affect ABCB1 and ABCG2 transport activity (Figure 8). Consistent with these findings, flunarizine also did not appreciably affect the plasma concentration and tumor accumulation of gefitinib in the PDX animal study (Table 5). Therefore, modulation of the efflux transporters ABCB1 and ABCB1 by flunarizine was not involved in the circumvention of gefitinib resistance.

**Table 5:** Flunarizine Did Not Affect the Bioavailability and Tumor Accumulation of Gefitinib in the PDX Model^a^

	gefitinib	gefitinib + flunarizine	gefitinib + SAHA
plasma concentration of gefitinib (nM)	32 ± 6	29 ± 4	33 ± 7
tumor accumulation of gefitinib (nmol/mg protein)	121 ± 16	135 ± 21	128 ± 11

a The blood sample was taken at 4 h after the last gefitinib dosing before animal sacrifice. Tumor xenograft was excised for drug content measurement after animal sacrifice. Reported findings represent mean ± standard deviation (*n* = 6).

Taken together, our findings provide a detailed mechanistic understanding about the drug repurposing use of flunarizine for circumvention of drug resistance via its novel HDAC inhibitory effect.

## Conclusions

Our study demonstrated that flunarizine could overcome gefitinib resistance both in vitro and in vivo, presumably by inducing apoptosis and retarding cell cycle progression via HDAC inhibition. As flunarizine is clinically approved with a favorable safety profile, our findings will pave the way for clinical evaluation of flunarizine to potentiate the anticancer efficacy of the EGFR TKI regimen.

## Materials and Methods

### Cell Lines and Chemicals

Human NSCLC cell lines A549, H820, H1650, H1975, and HCC827 were purchased from the American Type Culture Collection (ATCC) (Manassas, Virginia). The human bronchial epithelial cell line BEAS-2B (ATCC) was used to evaluate the general toxicity of the drug combination in normal lung tissues. The cell lines were maintained in Dulbecco’s modified minimal essential medium (A549 and BEAS-2B) and RPMI1640 medium (H820, H1650, H1975, and HCC827), supplemented with 10% fetal bovine serum, 100 μg/mL streptomycin, and 100 units/mL penicillin at 37 °C and 5% CO_2_.

Vorinostat (SAHA) and the repurposed drug candidates (including alfuzosin, baclofen, candesartan, cinnarizine, clemastine, danazol, disulfiram, flunarizine, guanfacine, raloxifene, telmisartan, and triamterene) were obtained from Cayman Chemical Company (Ann Arbor, Michigan).

### Cell Proliferation Assay and Drug Combination Analysis

Sulforhodamine B (SRB) assay was conducted to evaluate cancer cell proliferation as described previously.^42^ Briefly, cells ((3–6) × 10^3^ cells per well in 100 μL of culture medium) were seeded in a 96-well plate and allowed to equilibrate overnight. Then, the cells were treated with gefitinib alone, SAHA alone, HDACI candidate alone, or a combination of gefitinib + SAHA/HDACI candidate (in a fixed drug ratio) for 72 h. Afterward, the cells were fixed with 50 μL of trichloroacetic acid (50% w/v) at 4 °C for 1 h and then stained with SRB solution (0.4% w/v in 1% acetic acid; 50 μL/well) at room temperature for 1 h. The plates were then washed with 1% acetic acid and air-dried. Finally, 10 mM Tris–HCl buffer solution (200 μL/well) was added to dissolve the bound SRB dye. Absorbance of the resulting magenta coloration was measured at 570 nm.

If the HDACI candidate alone produced a complete concentration–cell growth inhibition curve, the combination of gefitinib and the HDACI candidate (in a fixed drug ratio) was analyzed by the median effect analysis.^43^ The combination index (CI) was calculated using software CompuSyn (Biosoft, Gambride, U.K.). CI < 1, =1, and >1 indicate synergistic, additive, and antagonist effects, respectively. If the HDACI candidate alone did not significantly inhibit cancer cell proliferation (i.e., IC_50_ was not achieved), the potentiation of gefitinib-induced cell growth inhibition by the HDACI candidate was calculated.

### Cell-Free Biochemical HDAC Activity Assay

Inhibition of total HDAC activity in the nuclear extract (H1975 cells) by the HDACI candidate was further evaluated using a fluorometric HDAC activity assay kit (Cayman Chemical) according to the manufacturer’s instruction. SAHA was used as a positive control for comparison. Briefly, the crude nuclear extract was harvested from H1975 cells with a nuclear extraction kit (Cayman Chemical). The crude nuclear extract was incubated with the tested drug candidates (at a range of different concentrations) at 37 °C for 30 min in the presence of a HDAC substrate. Next, a HDAC assay developer was added to the reaction mixture and incubated at room temperature (15 min) to generate a fluorophore, which was measured using a CLARIOstar Plus fluorescence plate reader (BMG Labtech, Cary, North Carolina) with excitation at 340–360 nm and emission at 440–465 nm. The HDAC activity of the nuclear extract samples was presented as % inhibition from the vehicle control. Inhibition of specific HDAC isoforms by the tested drug candidate was determined by repeating the HDAC activity assay with individual recombinant human HDAC1, 2, 3, 6, and 8 proteins (Creative Biomart, Shirley, New York).

### Quantitative Real-Time PCR

Total RNA was isolated from the cancer cells after drug treatment (flunarizine or SAHA, 24 h) using the TRIzol reagent (Invitrogen, Carlsbad, California). Reverse transcription was performed using 1 μg of total RNA sample with the PrimeScript First Strand cDNA Synthesis Kit (TaKaRa Biomedical Technology) at 37 °C for 10 min. The cDNA was then amplified by real-time PCR to determine the relative expression of p27^Kip1^ and BIM using the KAPA SYBR FAST qPCR kit (KapaBiosystems, Wilmington, Massachusetts) with a LightCycler 480 Instrument I instrument (Roche Applied Science, Indianapolis, Indiana). The house-keeping gene GAPDH was also measured for normalization. Specific primer sequences used in the PCR reactions are p27^Kip1^, forward 5′-GGTTAGCGGAGCAATGCG-3′ and reverse 5′-TCCACAGAACCGGCATTTG-3′; Bim, forward 5′-GGCCCCTACCTCCCTACA-3′ and reverse 5′-GGGGTTTGTGTTCATTTGTCA-3′; and GAPDH, forward 5′-ACCACAGTCCATGCCATCAC-3′ and reverse 5′-TCCACCACCCTGTTGCTGTA-3′. The PCR temperature program was set at 95 °C for 5 min, followed by 35 cycles of 95 °C for 10 s and 60 °C for 10 s. The fluorescent signal was detected at the end of the PCR elongation step of every cycle to keep track of the amount of amplified DNA. Δ*Ct* was calculated by subtracting the *Ct* of GAPDH from the *Ct* of Bim or p27^Kip1^. Fold change in gene expression was analyzed by the 2^–ΔΔ*Ct*^ method.

### Chromatin Immunoprecipitation (ChIP) Assay

ChIP assay was performed using our established protocol.^44^ Briefly, H1975 cells were treated with SAHA (1 μM) or fluorinarizine (10 μM) for 24 h. Then, formaldehyde (1%) was used to cross-link the nuclear proteins with DNA (10 min at 37 °C), which was quenched using 0.125 M glycine (5 min at ambient temperature) at the end of the incubation. The cells were then washed with ice-cold phosphate buffer saline containing sodium butyrate (5 mM) (Cayman Chemical), scrapped, and resuspended in a lysis buffer (5 mM HEPES, 85 mM KCl, 0.5% NP-40, pH 8.0) supplemented with the Halt protease inhibitor cocktail (Life Technologies, Grand Island, New York). Using a probe sonicator (Fisherbrand Model 120 Sonic Dismembrator, Suwanee, Georgia), the DNA–protein complexes were then sheared into 100–400bp DNA fragments (checked by agarose gel electrophoresis). Specific antibodies (AcH3 (Ac-K9,14 H3), HDAC1, E2F1, c-JUN, or the isotype control IgG; Cell Signaling Technology) were then used to pull down the DNA–protein complexes of interest at 4 °C overnight. The amount of antibodies used for each ChIP sample (1 × 10^7^ cells) is 2 μg. The immunoprecipitated DNA was resuspended in Tris-EDTA buffer (100 μL). The relative amount of immunoprecipitated DNA was compared by quantitative real-time PCR, using primers specific for the Bim (*BCL2L11*) or p27^Kip1^ (*CDKN1B*) promoter (*BCL2L11*): forward 5′-TAGGTGAGCGGGAGGCTAGGACA-3′, reverse 5′-GTGCAGGCTCGGACAGGTAAAGGC-3′^45^ and *CDKN1B*: forward 5′-CAACCAATGGATCTCCTCCT-3′, reverse 5′-GCCTCTCTCGCACTCTCAAA-3′.^41^ The amount of input DNA was also detected for normalization (10% of the total input used in PCR reactions). The Ct value from the real-time PCR analysis for the immunoprecipitated DNA was compared to that of the input DNA to calculate the fold enrichment of each immunoprecipitation.

### Western Blot Analysis

After the cancer cells were treated with the designated drug candidates, they were harvested in lysis buffer (0.05 M HEPES, 0.15 M NaCl, 2 mM EDTA, 10% v/v glycerol, and 1% v/v Triton X-100, pH 7.4) supplemented with protease and phosphatase inhibitors. Sodium dodecyl sulfate poly(acrylamide) gel electrophoresis (SDS-PAGE) was used to separate the different proteins in the whole cell lysates, which were then transferred to a poly(vinylidene difluoride) membrane for immunoblot analysis using different specific antibodies (acetylated histone H3, acetylated α-tubulin, histone H3, or GAPDH; Cell Signaling Technology). The bands corresponding to specific proteins of interest were visualized by using the WesternBright ECL HRP substrate (Advansta, San Jose, California). A chemiluminescent Western blot signal was detected and analyzed using a ChemiDoc MP system (Bio-Rad Laboratories, Hercules, California).

### Apoptosis Assay

Cancer cells were treated with gefitinib alone (0.2 or 5 μM), flunarizine alone (5, 10, or 20 μM), SAHA alone (0.5, 1, or 2 μM), or combinations of gefitinib and flunarizine/SAHA for 48 h. Following the drug treatment, the cells were harvested and stained using an Annexin V-FITC/7-AAD apoptosis kit (Elabscience, Houston, Texas) according to the manufacturer’s instruction. At least 10,000 events were acquired using a LSRFortessa cell analyzer, and the flow cytometric data were analyzed using BD FACSDiva software (BD Biosciences). Apoptotic cells were defined as the ones stained positive for both Annexin V-FITC and 7-AAD.

### Cell Cycle Analysis

The cell cycle profile of cancer cells was examined by a standard flow cytometry-based propidium iodide staining method after treatment with gefitinib alone or its combination with the repurposed drug candidates. HCC827 and H1975 cells were treated with gefitinib alone (0.01 or 0.05 μM in HCC827 cells or 5 or 10 μM in H1975 cells), flunarizine alone (5 or 10 μM), or a gefitinib–flunarizine combination for 24 h. The cells were then collected, resuspended in phosphate buffered saline, and fixed with 70% ice-cold ethanol overnight. Afterward, ethanol was discarded, and the fixed cells were incubated with RNase I (25 μg/mL) at 37 °C for 30 min. Then, the cells were stained with propidium iodide (50 μg/mL) at room temperature for at least 1 h and analyzed by using a flow cytometer (BD LSRFortessa; BD Biosciences). At least 10,000 events were acquired for each sample. ModFit software (Verity Software House, Topsham, Maine) was used to analyze the cell cycle profile.

### ABC Transporter-Mediated Drug Efflux Assay

As the two major efflux transporters (ABCB1 and ABCG2) are known to mediate the cellular efflux and drug resistance to gefitinib,^20^ flow cytometric assay was carried out to investigate the potential inhibition of ABCB1 and ABCG2 drug efflux activity by flunarizine in HEK293 cells stably transfected with the respective transporter as described previously.^46^ Cellular retention of fluorescent probe ABCB1 or ABCG2 substrates (rhodamine 123 (Rh123, 0.5 μg/mL) or pheophorbide A (PhA, 1 μM), respectively) in the presence or absence of flunarizine was determined. Specific inhibitors for ABCB1 (PSC833) and ABCG2 (FTC) were used as a control for comparison. Cells were analyzed by using a LSRFortessa cell analyzer (BD Biosciences). The fluorescent signal of Rh123 and PhA was detected with a 488 nm argon laser and a 670 nm bandpass filter. At least 10,000 events were collected for quantitative analysis.

### Wound Healing Assay

H1975 cells were seeded in 6-well plates for 24 h. After the cells had grown to about 80% confluence, a physical gap was created on the cell monolayer by scratching with a pipet tip. Afterward, the cells were incubated with flunarizine or culture medium (control) for another 48 h. Images of the cells around the gap were captured using a light microscope at 24 and 48 h. The scratch closure rate was estimated by the change in the wound width over time. It was presented as the relative gap width (%) by dividing the average distance between the two margins of the scratch at the indicated time by the initial distance at the start of the wound healing assay.

### Cell Migration Assay Using Transwell Inserts

The migration of H1975 cells was studied in transwell chambers (10 mm tissue culture transwell inserts with an 8-μm pore size polycarbonate membrane, 24-well companion plate, BD Biosciences, Bedford, Massachusetts). H1975 cells (5 × 10^5^) were seeded into each well of the upper chamber in RPMI 1640 culture medium (without fetal bovine serum) containing 0, 5, or 10 μM flunarizine. The culture medium (500 μL) supplemented with 10% fetal bovine serum was placed in the lower chamber. Noninvading cells that remained on the top side of the transwell membrane were removed after 48 h of incubation. The cells that invaded across the transwell membrane were attached to the lower surface of the transwell membrane. They were fixed with paraformaldehyde (4%, w/v) at room temperature for 30 min and stained with crystal violet (0.4%, w/v). Random fields were counted, and representative pictures were captured with a light microscope.

### Patient-Derived Tumor Xenograft

The antitumor activity of the gefitinib–flunarizine combination was further evaluated in NOD SCID γ (NSG) mice bearing patient-derived tumor xenograft (PDX).^47^ Tumor specimens were collected from an Asian NSCLC patient refractory to first-generation EGFR-TKI (erlotinib) with informed consent. The collection and use of patient-derived tumor specimens for research were approved by the Research Ethics Committee (Kowloon Central/Kowloon East) (reference number: KC/KE-17-0229/ER-1). Demographic information on the relevant NSCLC patient is summarized in Table 4. The animal experimental work was approved by the CUHK Animal Experimentation Ethics Committee (approval number 20-009-HMF). Briefly, the patient tumor tissue was implanted subcutaneously into the flanks of a NSG mouse to develop the first passage PDX tumor. It was then excised, chopped down into small pieces, and dissociated into a cell suspension that was subcutaneously injected into the flank of other NSG mice (18–24 g). A total of 36 mice bearing PDX were successfully established. When PDX tumors become palpable approximately 2 weeks afterward, treatment was commenced in the following treatment groups (*n* = 6 per group): (i) vehicle control; (ii) gefitinib alone (50 mg/kg/day); (iii) flunarizine alone (40 mg/kg/day); (iv) gefitinib (50 mg/kg/day) + flunarizine (40 mg/kg/day) combination; (v) SAHA alone (40 mg/kg/day); and (vi) gefitinib (50 mg/kg/day) + SAHA (40 mg/kg/day) by oral gavage daily for 32 days. A Vernier caliper was used to measure the tumor size, and tumor volumes were estimated using the formula tumor volume = 1/2 × (shortest diameter)^2^ × (longest diameter). Tumor growth was monitored until day 32. Animal mortality and morbidity were monitored daily. Drug toxicity was monitored in tumor-bearing mice using two parameters: (i) lethal toxicity (defined by any death in drug-treated mice occurring before any death in control mice) and (ii) % body weight loss. At the end of the drug treatment and observation period, the experimental animals were sacrificed by cervical dislocation.

### Determination of Plasma Concentration and Tumor Accumulation of Gefitinib Using Liquid Chromatography with Tandem Mass Spectrometry (LC-MS/MS)

A LC-MS/MS system (consisting of an Agilent 6430 triple quadrupole mass spectrometer equipped with an electrospray ionization source, an Agilent 1290 pump, and an autosampler (Agilent Technologies Inc., Santa Clara, California)) was used to analyze the plasma and tumor samples according to the protocol adopted from Guan et al. with minor modification.^48^ A Zorbax Eclipse XDB-C_18_ column (4.6 × 250 mm^2^, 5 μm, Agilent Technologies) was used to achieve chromatographic separation of the analytes. An isocratic chromatographic system was used. The mobile phase was water:acetonitrile (35:65, v/v) with 0.1% formic acid at a flow rate of 0.25 mL/min. The injection volume of the sample was 10 μL. The triple quadrupole mass spectrometer was operated in positive electrospray ionization mode. The spray voltage was +3000 V, and the vaporizer temperature was 280 °C. The pressure of the collision gas was 1.0 mTorr. The collision energies for gefitinib and vatalanib (internal standard) were set at 24 and 33 eV, respectively. The quantification of gefitinib was determined using selected reaction monitoring (SRM) *m*/*z* 447.6 →  128.1 for gefitinib and *m*/*z* 347.1  →  311.1 for vatalanib (internal standard). On the last day of the treatment period (day 32), blood was drawn before animal sacrifice at 4 h after the last gefitinib dosing (∼*t*_max_ of gefitinib) for the measurement of the plasma gefitinib concentration. Tumor xenografts were also excised for determination of the gefitinib content by LC-MS/MS.

### Statistical Analysis

All experiments were conducted at least three times. Data were presented as the mean ± standard deviation (SD). Two-tailed Student’s *t*-test was used to statistically compare two experimental groups. One-way analysis of variance (ANOVA) followed by Tukey’s multiple comparison test was used to compare three or more groups. *P < 0.05 and **P < 0.01 were considered statistical significant.

## Supporting Information Available

Cytotoxicity of flunarizine or its combination with gefitinib on the human bronchial epithelial cell line BEAS-2B (PDF)

## Notes

All relevant data are within the paper.

## Notes

**Ethics Statement** The collection and use of patient-derived tumor specimens for research were approved by the Research Ethics Committee (Kowloon Central/Kowloon East) (reference number: KC/KE-17-0229/ER-1). The animal experiment protocol was approved by the CUHK Animal Experimentation Ethics Committee (approval number 20-009-HMF).

## Abbreviations

ChIP: chromatin immunoprecipitation
CI: combination index
EGFR: epidermal growth factor receptor
HDACI: histone deacetylase inhibitor
NSCLC: non-small cell lung cancer
PDX: patient-derived tumor xenograft
SAHA: vorinostat
SRB: sulforhodamine B
TKI: tyrosine kinase inhibitor
