# Supplementary material for: Circumvention of Gefitinib Resistance by Repurposing Flunarizine via Histone Deacetylase Inhibition
Source: ACS Pharmacol Transl Sci. 2023 Sep 28;6(10):1531–43. doi: 10.1021/acsptsci.3c00202 (PMC10580381; doi:10.1021/acsptsci.3c00202)
Supplement: Supplementary file 1 — pt3c00202_si_001.pdf [file pt3c00202_si_001.pdf]

Supporting Information

Circumvention of gefitinib resistance by repurposing flunarizine via histone deacetylase inhibition

Kenneth K.W. To[1,*], James C.H. Chow[2], Ka-Man. Cheung[2], William C.S. Cho[2]
[1]School of Pharmacy, Faculty of Medicine, The Chinese University of Hong Kong, Hong Kong SAR, China
[2]Department of Clinical Oncology, Queen Elizabeth Hospital, Hong Kong SAR, China

*Corresponding author: Kenneth K.W. To, School of Pharmacy, Room 801N, Lo Kwee-Seong Integrated Biomedical Sciences Building, The Chinese University of Hong Kong, Area 39, Shatin, New Territories, Hong Kong SAR, China; Phone: (852) 39438017; Fax: (852) 26035295; Email: kennethto@cuhk.edu.hk

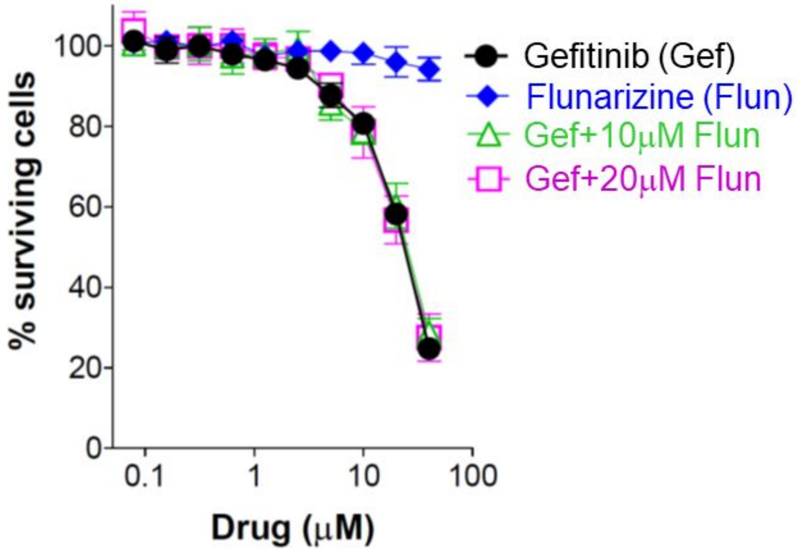

Supp. Fig. 1

Supp. Fig. 1. Cytotoxicity of flunarizine or its combination with gefitinib on a human bronchial epithelial cell line BEAS-2B. Cells were treated with gefitinib alone, flunarizine alone, or combination of gefitinib (serial dilution: 0.3125 – 40 μM) and flunarizine (fixed concentration of 10 μM or 20 μM) for 72 h. Cell proliferation was measured by sulforhodamine assay.
